# Inflammatory Cardiomyopathy: A Current View on the Pathophysiology, Diagnosis, and Treatment

**DOI:** 10.1155/2016/4087632

**Published:** 2016-06-12

**Authors:** Jan Krejci, Dalibor Mlejnek, Dana Sochorova, Petr Nemec

**Affiliations:** ^1^Department of Cardiovascular Diseases, St. Anne's University Hospital-International Clinical Research Center, Pekarska 53, 65691 Brno, Czech Republic; ^2^International Clinical Research Center and Masaryk University, Brno, Czech Republic; ^3^Centre for Cardiovascular Surgery and Transplantation, Brno, Czech Republic

## Abstract

Inflammatory cardiomyopathy is defined as inflammation of the heart muscle associated with impaired function of the myocardium. In our region, its etiology is most often viral. Viral infection is a possible trigger of immune and autoimmune mechanisms which contributed to the damage of myocardial function. Myocarditis is considered the most common cause of dilated cardiomyopathy. Typical manifestation of this disease is heart failure, chest pain, or arrhythmias. The most important noninvasive diagnostic method is magnetic resonance imaging, but the gold standard of diagnostics is invasive examination, endomyocardial biopsy. In a significant proportion of cases with impaired left ventricular systolic function, recovery occurs spontaneously in several weeks and therefore it is appropriate to postpone critical therapeutic decisions about 3–6 months after start of the treatment. Therapy is based on standard heart failure treatment; immunosuppressive or antimicrobial treatment may be considered in some cases depending on the results of endomyocardial biopsy. If severe dysfunction of the left ventricle persists, device therapy may be needed.

## 1. Introduction

**Figure 1:**
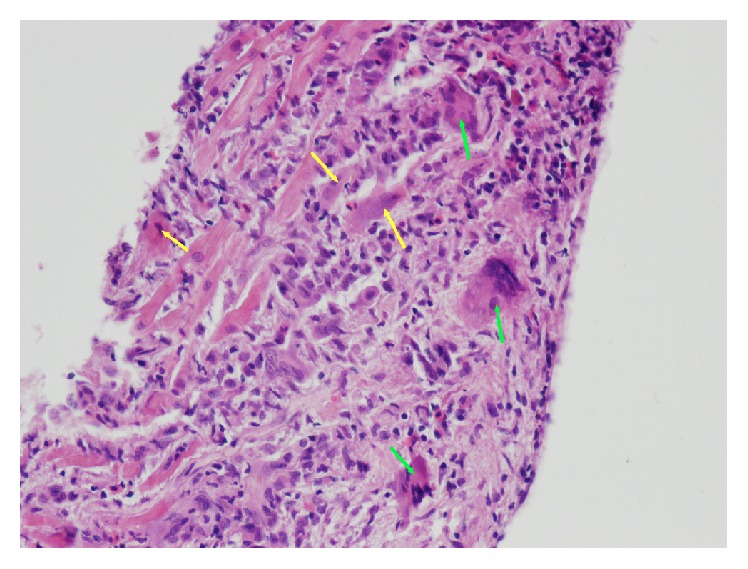
Giant cell myocarditis, hematoxylin eosin, magnification 200x. Massive inflammatory myocardial lesions with regressive cardiomyocytes (yellow arrows) and mixed reactive cellulisation with the giant multinuclear elements (green arrows) (from the archive of V. Zampachova, MD).

Inflammatory cardiomyopathy (ICM) is defined as inflammation of the heart muscle associated with impaired function of the myocardium, which has most often the morphology of dilated cardiomyopathy. Inflammation of the heart muscle itself, that is, myocarditis, may have many infectious (viral, bacterial, and protozoal infections) and noninfectious causes (e.g., myocarditis accompanying autoimmune disease or hypersensitivity to certain noxious substances). According to the 1995 WHO/ISCF definition, myocarditis is an inflammation of the heart muscle and is diagnosed by using histological, immunological, and immunohistochemical criteria [1]. In 2013, the Position Statement of the European Society of Cardiology Working Group on Myocardial and Pericardial Diseases was published. It is stressed that histological and immunohistochemical evidence of myocardial inflammation is absolutely crucial, and therefore endomyocardial biopsy (EMB) is necessary for the final* in vivo* confirmation of myocarditis. Assessment of the bioptic samples of the myocardium allows beside the diagnosis of myocarditis itself also its accurate classification by typing of infiltrating cells or histological character of lesions (e.g., lymphocyte or eosinophilic infiltration, giant cell myocarditis (GCM) (see Figure 1), granulomatous or necrotizing process, and autoimmune features) with all important prognostic and therapeutic consequences. An integral and key part of EMB samples evaluation is the search for potential infectious agents in the myocardium, usually using reverse polymerase chain reaction (PCR) [2].

*Clinical picture* of myocarditis can vary, which may bring difficulties in the diagnosis of this disease, but it has been shown that the most frequent manifestation is heart failure [3].

It usually occurs due to a dysfunction of the left ventricle (LV), which is an integral part of the diagnosis of inflammatory cardiomyopathy.

The most common etiological cause of myocarditis in Western civilization is considered to be viral infection. In recent decades there has been a shift in viral spectrum; previously dominating adenovirus and enteroviruses were currently replaced by parvovirus B19 (PVB19) and human herpes virus 6 (HHV-6) [4]. This has been also convincingly confirmed by the results coming from the Marburg Registry, the largest database of patients with suspected myocarditis who underwent EMB [5].

In Central and South America, Chagas disease is often found. It is caused by the protozoan* Trypanosoma cruzi* and one of the disease symptoms is myocarditis [6]. In some endemic regions,* Borrelia burgdorferi* is relatively frequently detected in patients with myocarditis [7, 8].

Contemporary view on the* pathophysiology* of myocarditis is based on animal models of enteroviral myocarditis and assumes the three-phase evolution of the disease [9–11]. The first acute phase is associated with viral entry into myocytes over the virus-specific receptor (CAR coxsackie-adenoviral receptor) with the participation of coreceptors (DAF, decay accelerating factor, for enteroviruses and integrins *ανβ* 3 and 5 for adenoviruses) [12]. In this phase, which lasts several days to weeks, viral replication and inflammatory mediators production associated with nonspecific immunity are predominantly responsible for myocytes impairment (and thus the function of the myocardium). In clinical practice, this period may often be asymptomatic. The second phase starts usually 2–4 weeks after onset of the disease and is characterized by a specific immune reaction. This includes both cellular and antibody-mediated immune response which often could have autoimmune features. These autoimmune reactions are based on two main mechanisms: the first is the cross-reactivity of viral epitopes and some cardiac structures (molecular mimicry phenomenon); another option is the exposure of originally intracellular structures to the immune system that occurs after the virus-induced damage of myocytes. Such a situation is seen in the production of antibodies against alpha and beta myosin-heavy-chains, wherein the antibody against alpha chains is considered organ (heart) specific. Antibodies against myosin have a negative effect on myocyte contractility, which was confirmed* in vitro* and also in animal experiments. They also affect calcium channels, leading to calcium overload of myocytes. In patients with ICM, a number of other antibodies was captured, for example, antibodies against beta-adrenoceptors, against M2 muscarin-receptors, or against troponin [5, 13–16]. The third phase of the disease occurs after several weeks or months and may include either retreat of inflammation and improvement in LV function (in 50–70% of cases, usually after removal of viruses from myocardium) or persistent LV dysfunction associated with development of postinflammatory dilated cardiomyopathy (DCM). A number of factors play an important role in the disease course, for example, degree of initial damage of the myocardium, the intensity and duration of inflammation, or the persistence of viral replication [17, 18].

Whether the described course takes place in every case of myocarditis caused by various viruses (i.e., those that are primarily not invading myocytes, but endothelial cells of blood vessels as is the case of PVB19 or HHV6 infection) is not entirely clear [19]. It seems likely that a necessary condition for the creation of myocarditis is certain genetic predisposition; the vast majority of individuals will not develop myocarditis, even after meeting the so-called cardiotropic virus. This theory is also supported by more frequent occurrence of myocarditis in some families [2, 11].

*Epidemiology*. The real incidence of myocarditis is difficult to determine exactly due to the complex definitive diagnosis in routine clinical practice. In young adults who died suddenly, myocarditis was found* post mortem* in a wide range between 2 and 42%; other studies indicate up to 46% incidence of myocarditis in children with unexplained DCM [2]. Previous works using the Dallas Criteria reported incidence of biopsy-proven myocarditis in 9–16% of DCM cases [20]. More recent studies [21, 22] demonstrate that almost 50% of patients with clinical diagnosis of DCM have immunohistochemically detectable myocarditis and thus could be classified as ICM. Myocarditis (or ICM in particular) is considered as the most common cause of dilated cardiomyopathy [23].

Another interesting fact is the frequent detection of viral nucleic acids in the myocardium (up to 60–80% of cases) [21, 22, 24]. Given the fact that some viruses (e.g., PVB19) are often found even in individuals with normal LV function, their real significance is not elucidated with certainty, and this issue is the subject of intense research [24–27]. According to some authors, the presence of any virus in the myocardium is a negative prognostic factor [28, 29], while others have not confirmed that presence of a virus has negative effect on the prognosis and evolution of LV function [22, 30]. A lot of controversies are about the most frequently detected PVB19 or HHV6, respectively, because not only is their presence in the myocardium that plays an important role in the pathogenicity, but there are also other factors such as viral load, active virus replication, coinfection with other viruses, genetic background, sex differences, and others influencing their etiological role [31–35]. In Marburg Registry comprising data from almost 12,500 patients it was shown that prevalence of PVB19 in patients with myocardial inflammation and LV dysfunction was higher than in the group with inflammation and preserved LV function. The same was true for comparison of patients with LVEF below 45% with and without myocarditis (PVB19 presence in 33.3% versus 17.6%; *p* < 0.05) [5].

## 2. Clinical Manifestation

Clinical manifestation of myocarditis/inflammatory cardiomyopathy ranges from virtually asymptomatic course of a mildly ongoing disease with slightly lesser extent of impaired myocardium on one hand and severe fulminant heart failure accompanied with malignant arrhythmias on the other. Sometimes, sudden cardiac death may be the first manifestation of the disease in a previously completely healthy individual. Often mentioned initial viral infection foregoing viral/postviral myocarditis can pass subclinically; therefore its absence in history of the patient certainly does not rule out possible evolution of inflammatory myocardial damage. In milder cases, of course, the symptoms during the active phase are less dramatic, but it does not automatically mean better long-term prognosis. However, extent of myocardial damage in acute phase is one of the important factors determining recovery of LV function in the later period [2, 5, 9–11, 17, 18].

**Figure 2:**
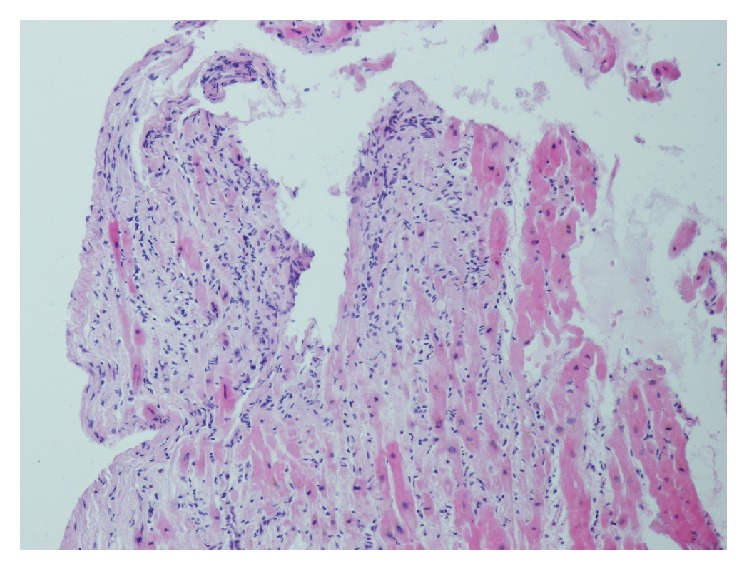
Fulminant myocarditis, hematoxylin eosin, magnification 100x. Residual cardiomyocytes with fibrotisation and dense lymphocytic cellulisation (from the archive of V. Zampachova, MD).

**Figure 3:**
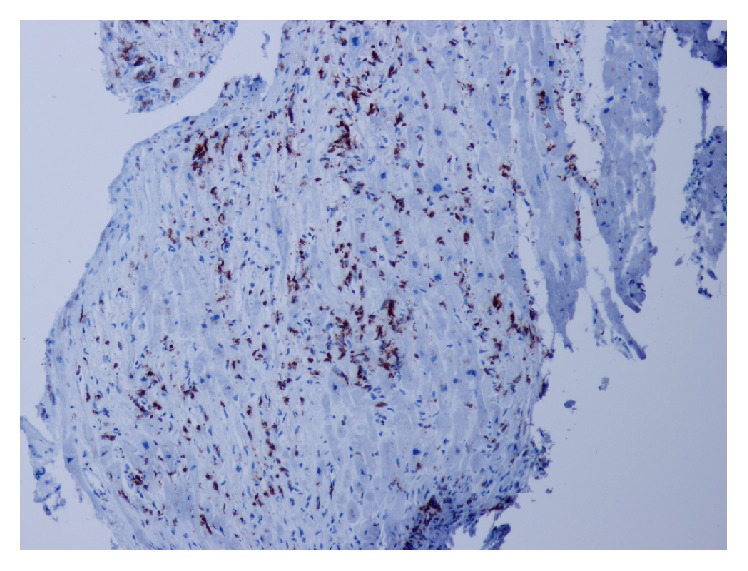
Fulminant myocarditis, detection of CD3+ T lymphocytes, immunohistochemistry, magnification 100x. Numerous positive elements (dark nuclei), focally detected 250 CD3+ T cells/mm^2^ (from the archive of V. Zampachova, MD).

The most common manifestation of myocarditis is* heart failure*. It may have a gradual onset and only mild symptoms, but there is no exception to see rapidly emerging cases terminating in cardiogenic shock, where only the implantation of mechanical circulatory support device or urgent heart transplantation can save the patient's life. This scenario is typical for fulminant myocarditis (see Figures 2 and 3); if the patient survives the acute phase, a significant improvement or even a complete normalization of LV systolic function with very good long-term prognosis may occur in a few weeks [36]. This is true to certain degrees for all types of myocarditis with initial systolic dysfunction, when LV function improves spontaneously or after standard heart failure treatment at least in half of the cases [17–20]. It is therefore appropriate to postpone crucial therapeutic decisions (such as implantation of a cardioverter/defibrillator with/without resynchronisation function or heart transplantation) for a period after the acute phase (when this is allowed by clinical situation), which means in clinical practice delay of about 3–6 months after disease onset or after the start of the treatment.

Our data from 6-month follow-up showed that the retreat of inflammatory infiltration in the myocardium is associated with improvement of a number of echocardiographic parameters, with decrease of NTproBNP and improvement of the functional status [37]. Tschöpe et al. published an interesting study describing a high incidence of PVB19 in patients with isolated diastolic dysfunction of LV (in 95% of patients), while in the group with normal diastolic function PVB19 occurred only in 24% of patients. So not only the systolic, but the diastolic dysfunction as well may be associated with viral heart disease or myocarditis. The basis of this fact seems to be the presence of endothelial dysfunction in individuals with endothelial cells infection, which may also be associated with a higher incidence of chest pain as a clinical manifestation of the disease [38]. The urgency of EMB in cases of isolated diastolic dysfunction is questionable, because there are no data for introduction of specific therapy according to the bioptic result regarding the presence of myocarditis. Thus, another clinical scenario is the manifestation of the disease by* chest pain* that can mimic angina pectoris or may have a pericarditis-like character, particularly if perimyocarditis is present. Patients are often brought to the catheterization laboratory to rule out acute coronary syndrome (especially if elevation of markers of myocardial damage is present). Normal findings on coronary arteries and the exclusion of other pathology (e.g., aortic dissection, atrial or ventricular tachycardia, but also noncardiac involvement in florid gastroduodenal ulcer disease or severe anemia) in such cases lead to consideration of possible myocarditis [39].

Third dominating complaint that brings a patient to the physician can be symptoms related to* arrhythmias*. Arrhythmias may be both supraventricular and ventricular; conduction disturbances or serious ventricular arrhythmias suggesting the possibility of giant cell myocarditis, cardiac sarcoidosis, or* Borrelia burgdorferi* associated myocarditis. Myocarditis can also be found incidentally at autopsy in patients who died suddenly, probably on the basis of malignant arrhythmia. Fortunately, we see more often less dramatic course with the presence of palpitations, dizziness, or even syncope, which always have to alert attending physician to the possible presence of serious arrhythmias.

Of course, it is not unique that all described symptoms may be present in one patient, either simultaneously or at different time phases of the disease. In terms of prognosis, it has been reported that cases with symptoms of heart failure, namely, those which meet the criteria of inflammatory cardiomyopathy, have a poorer prognosis than cases manifesting by chest pain or arrhythmias [28].

Another disease that should be mentioned while speaking about ICM is peripartum cardiomyopathy (PPCM) [40–42]. PPCM is manifested by systolic heart failure in previously healthy women at the end of pregnancy or in the first months after the birth. The causes of the disease are not definitively clarified, but according to some of the authors myocarditis could play an important role in pathogenesis of this quite mysterious disease [43, 44]. It affects more often African-American women (relative risk is almost 16 times higher) [45] and has a relatively high frequency of recovery of LV function (on the contrary, particularly among white women) [46, 47]. Nevertheless, in about 10% of women, it progresses to severe heart failure, when the only solution may be urgent heart transplantation or LVAD implantation. In less developed countries where these treatment options are not available (and where PPCM is unfortunately relatively more frequent), the mortality is in comparison with European countries and the USA not negligible [47–49].

## 3. Diagnostics

In the past, the* diagnostics of myocarditis* was a difficult and challenging task. Even today, despite various imaging modalities that are available nowadays myocarditis often remains a diagnosis* per exclusionem*. The Position Statement of ESC Working Group on myocardial and pericardial diseases based clinical suspicion for myocarditis on the presence of typical clinical presentation (heart failure, chest pain, and arrhythmia) and noninvasive imagining techniques (see Diagnostic Criteria for Clinically Suspected Myocarditis) [2]. Endomyocardial biopsy is recommended for all patients who fulfil clinical diagnostic criteria and remains the standard tool for definitive confirmation of the diagnosis [2, 5, 10, 11, 17, 19]. However, this procedure is the method of first choice only in specialized centers with experience in performing EMB with advanced laboratory equipment needed for complex evaluation of EMB samples.

*Diagnostic Criteria for Clinically Suspected Myocarditis*. Diagnosis of myocarditis is suspected in presence of

- ≥1 clinical presentation and ≥1 diagnostic criterion,
- ≥2 diagnostic criteria, if the patient is asymptomatic.

*Clinical Presentation*. Clinical presentation involves

- chest pain,
- acute or chronic heart failure,
- arrhythmic symptoms (palpitations, syncope, and sudden cardiac death).

*Diagnostic Criteria. *Diagnostic criteria are as follows:

- Electrocardiogram* (ECG) *test features (atrioventricular block, bundle branch block, ST/T-wave changes, supraventricular or ventricular arrhythmias, low voltage of QRS complex, and abnormal Q waves).
- Markers of myocardial necrosis (elevated cardiac troponins or CK-MB).
- Functional and structural abnormalities on echocardiography or CMR imaging (impaired left or right ventricle function, with or without left or right ventricle dilatation, increased ventricle wall thickness, pericardial effusion, and intracardiac thrombi).
- Tissue characteristics by CMR (presence of at least two of three Lake Louise criteria, myocardial oedema and early and late gadolinium enhancement).

Similarly to diagnostics of other diseases in cardiology, the process starts with simply conventional examinations such as* ECG*, which can have very variable and also nonspecific findings (presence of arrhythmias, changes of PQ and ST interval, prolongation of QRS complex, and the presence of Q waves), although some findings (especially the presence of rhythm disorders, i.e., ventricular tachycardia or atrioventricular block of 2nd or 3rd degree) may be suggestive of special types of myocarditis (giant cell myocarditis or cardiac sarcoidosis).

Another basic diagnostic method is* echocardiography*. Here as well, there is not any typical finding allowing diagnosis with some of nonspecific echocardiographic features including both global and regional kinetic disorders of the left or right ventricle, diastolic dysfunction, left ventricle hypertrophy, and pericardial effusion. But even a normal finding does not rule the diagnosis out. The value of echocardiography lies rather in excluding other causes of the symptoms (valvular or pericardial disease, aortic dissection) and also in risk stratification based on evaluation of left ventricle systolic dysfunction [2, 9, 11, 17].

The most important noninvasive diagnostic method is* magnetic resonance imaging* (MRI) which is a routinely available technique in last years and is suited for the evaluation of both morphological and functional myocardial impairment and tissue characterization [50–53]. The clinical suspicion of myocarditis is one of the most common indications for MRI study in cardiology because it is an accurate modality for the assessment of a number of common features in myocarditis: myocardial oedema and hyperemia, capillary leak, necrosis and fibrosis, and contraction abnormalities or pericardial effusion [54]. The Lake Louise criteria have been proposed to standardize the evaluation of findings and to improve the diagnostic accuracy [50]. The criteria are based on the evaluation of myocardial oedema (T2-weighted sequences) frequently present in acute inflammation, early gadolinium enhancement (EGE) related to hyperemia, and in particular the assessment of the presence of the late gadolinium enhancement (LGE) with the presence of a characteristic type of gadolinium accumulation in areas of myocardial necrosis or fibrotic reparative changes. If two of these three criteria are present, the MRI imaging demonstrates 67% sensitivity, 91% specificity, and 78% diagnostic accuracy [2, 50]. The LGE was shown as important for prognostic stratification; if not present, the outcome is very good; on the contrary the presence of LGE is considered to be a significant predictor of overall and cardiovascular mortality (OR 8.4 and 12.8, resp.) [55].

The diagnostic sensitivity of MRI is higher in acute scenarios than in chronic cases with less intensive inflammatory changes. The sensitivity is higher also in cases with clinical manifestation by chest pain (“infarction-like” symptoms) than in patients with arrhythmias or heart failure [56]. Despite several technical difficulties in evaluation of, for example, early gadolinium enhancement, MRI is definitely one of the leading diagnostic modalities if myocarditis is suspected. However, especially in fulminant forms, MRI should not delay EMB performance representing the gold standard with more significant additive information regarding treatment decision [2, 19, 34].

Some of the* laboratory tests* may be useful in myocarditis diagnostics; most useful is detection of myocardial damage in acute phase by troponin and CK-MB elevation; elevation of troponin was identified as a negative prognostic factor and may be also used for long-term monitoring of disease activity [57]. Elevated levels of natriuretic peptides are neither diagnostic nor specific but they can identify patients with worse prognosis [58]. Also the detection of certain antibodies against the myocardial structures (see above) related to autoimmune impairment showed to be contributive to diagnostics but standardized commercial kits are currently not available [15, 19]. The presence of antibodies could be one of the markers of positive response to immunosuppressive treatment [59]. If antibodies are detected in healthy relatives of patients with dilated cardiomyopathy, the risk of disease manifestation in these individuals is higher [2, 15]. Inflammatory markers can be elevated but this is not a rule. The diagnostic approach based on serological tests from peripheral blood often used in the past did not show significant correlation with EMB results [60].

Recently, the development of new sophisticated methods highlights the tendency for less invasive or even noninvasive diagnosis of myocarditis using modern approaches. One of these methods is detection of different gene transcription which seems to be promising due to high specificity and sensitivity to distinguish myocarditis and dilated cardiomyopathy [61, 62]. Another method is evaluation of the miRNA levels. MiRNAs are small noncoding RNAs regulating posttranscription gene expression. Their levels differ in various physiologic and pathologic conditions and the first studies based on animal models showed that some of them are upregulated (e.g., miRNA-155, miRNA-146b, and miRNA-21) in myocarditis and can distinguish inflammatory and noninflammatory myocardial impairment [63]. Similar upregulation was also proved in patients with viral myocarditis for miRNA-155 and miRNA-148a [64]. Different expression of miRNAs [65] and different gene transcription [66] were recently published comparing individuals with replication active and latent myocardial PVB19 infection. The PVB19 replication activity seems to be the crucial factor in the understanding of PVB19 role in pathogenesis of myocarditis. The study by Kühl et al. including 415 patients with the PVB19 myocardial presence showed that only in 15,9% patients the virus was replicating and it was in relation to changes in cardiac gene expression, for example, INF- *β*1 (up-regulation), FOXP3, ADIPOR2, and IL-10 (downregulation), and with elevated mRNA levels. These methods could be used for prognostic stratification and personalised treatment decisions [66].

*Coronary angiography* is indicated to exclude coronary artery disease (CAD) as one of the possible causes of the symptoms and should be done in all patients in risk of CAD regardless of the symptoms, which means also in patients without chest pain.

*Endomyocardial biopsy* is still considered as gold standard and the only method for definitive diagnosis* in vivo*. The sample can be obtained from left or right ventricle (or both); the diagnostic yield probably depends on the number of samples, not on the particular site of EMB [2, 67] despite the fact that some studies showed higher sensitivity in left ventricular and biventricular biopsy than only right ventricular one [68, 69]. Unlike the presence of infiltrating cells, which is comparable in both ventricles, some characteristics differ between the two chambers; for example, degree of fibrosis is more pronounced in LV [67].

The endomyocardial biopsy use in diagnosing of myocarditis is not a completely new trend. The beginning dates back to the 80s when Dallas criteria were set to standardize the histology evaluation of biopsy samples [70]. When these criteria were found to be of low sensitivity and high interobserver variability on histology assessment, it was necessary to set new, more sensitive and precise criteria that could be used in routine practice [71, 72]. There is also a noticeable difference in indication of the EMB between the US and European countries. In the US, it is recommended to use EMB only in specific clinical scenarios [73, 74]; the ESC recommended approach is, however, more aggressive and EMB should be performed in all cases when myocarditis is clinically suspected [2]. The consensus of European pathologists published in 2013 also came to the same conclusion [75]. The addition of immunohistochemistry used for typing of infiltrative leucocytes constitutes a breakthrough due to higher sensitivity of EMB for detection of myocarditis [72]. At the turn of the millennium, Marburg's criteria were set and were based on the presence of more than 14 mononuclear leucocytes/mm^2^ of bioptic sample [5, 76]. The inclusion criteria of the TIMIC study added the alternative presence of more than 7 T-lymphocytes per mm^2^ as a second criterion [77]. The current position statement requires the simultaneous presence of both and, moreover, excluded patients with the presence of more than 4 monocytes per mm^2^ [2]. Recent studies from Berlin showed that setting novel cut-off values for the number of the infiltrating cells (e.g., more than 10 CD3+ cells per 10 mm^2^ or more than 30 CD45+ per mm^2^) could make the prognostic stratification even more precise. Another new approach is evaluation of perforin-positive cells; the presence of more than 2,95 cells/mm^2^ is related to a poor outcome [78]. The other immunohistochemical marker that can be also used is the assessment of HLA expression which upregulated during myocardial inflammation; this criterion was used for the selection of patients in Wojnicz et al.'s study [79]. We should be aware of a potential sampling error due to focal myocardial cellular infiltration which decreases the sensitivity of EMB [80, 81]. This could be sorted out by combination with the assessment of HLA antigen expression that is usually more diffuse. However, it is a semiquantitative method based on subjective assessment by the pathologist.

The evaluation of the samples should always include the evaluation of viral (or other agents) presence or more precisely the viral nucleic acid presence. PCR is the most common method used for viral detection in myocardium. Especially in PVB19 presence, the quantitative assessment of viral load (number of viral copies) should be done because low viral load might not be related to inflammation induction [31, 33]. Other authors consider the viral load as not so important and stress the need for replication activity evaluation (by detection of mRNA, miRNA profile, or gene transcription), which is of special interest in PVB19 where not replicating virus could be rather an “innocent bystander” than the direct cause of acute inflammation [65, 66].

From the foregoing facts can be concluded that the setting of diagnostic criteria is still in evolution and it can be assumed that it will lead to their further modifications in the future depending on new findings.

## 4. Therapy

The problem of therapeutic recommendations, or rather the reason why they are so cautiously formulated, is the fact that they are based more on results of small monocentric studies and institutional registries, while data from the randomized, multicenter, placebo-controlled trials are either very subtle or even completely absent [2, 5, 9–11, 17, 19, 22].

There is consensus on regime measures limiting physical activity for 6 months or till retreat of the inflammation in control EMB and/or till restitution of LV function [2]. Pharmacotherapy of inflammatory cardiomyopathy with the presence of LV dysfunction is based on administration of standard heart failure treatment according to current guidelines, consisting mainly of angiotensin converting enzyme inhibitors (ACEIs)/angiotensin receptor blockers (ARBs), beta-blockers, and aldosterone antagonists [82, 83]. For these drugs we also have some experimental and clinical data documenting the potential positive influence on inflammatory changes and the prognosis of patients [84–88]. Conversely administration of nonsteroid anti-inflammatory drugs (NSAIDs) and digoxin is not recommended as a result of animal experiments where these drugs have led to deterioration of LV function. Also, administration of positive inotropic agents may lead to further impairment of the myocardium already damaged by inflammation and should be reserved only for very exceptional situations [17].

In critical cases it is necessary to use a mechanical circulatory support, either as a “bridge to decision” or as a “bridge to transplantation” which may be in cases of persistent severe refractory heart failure the last therapeutic option. Approach to the treatment of arrhythmias and device therapy especially in primary prevention of sudden cardiac death should be preferably restrained in the acute phase because significant improvement in LV function and retreat of arrhythmias associated with regression of myocardial inflammation may be often seen in a few weeks. To overcome this critical acute phase, it is possible to use special external defibrillation equipment such Life-vest [2] in some countries. This can prevent the implantation of endovascular/intracardial devices for patients with only temporary need of antiarrhythmic nonpharmacologic treatment. Otherwise, hospitalization with monitoring of heart rhythm and evaluation of the arrhythmogenic risk with optional next therapeutic steps may be necessary.

In the specific treatment of myocarditis, the situation is ambiguous. For some specific subtypes of myocarditis, immunosuppression is associated with a distinct profit and is considered to be clearly indicated; this is especially the case of GCM [89–91], followed by eosinophilic myocarditis [92, 93]; immunosuppression should be started also in cardiac sarcoidosis [94]. Immunosuppressive schemes vary among different types of inflammation, in the case of GCM, immunosuppression should be far more aggressive, so this is why it is important to differentiate these types of myocarditis. In patients with chronic lymphocytic myocarditis with symptoms longer than six months, there are data from two randomized clinical trials showing the additive positive effect of combined immunosuppressive therapy (combination of prednisone and azathioprine) on echocardiographic parameters compared to standard care [77, 79].

In each of these studies a different dose was used and the duration of treatment was also different, although the same drugs were administered. In Wojnicz et al.'s study enhanced expression of HLA antigens was used as an inclusion criterion and, moreover, the presence of microbial agents in the myocardium was not ruled out [79]. Frustaci et al. included patients into the study according to the number of infiltrating cells and the absence of an infectious agent in the myocardium [77, 95]. Thus, these studies are not entirely consistent in methodology and therefore results cannot be simply “added up.” In addition, because both are single-center trials it would be required to verify the results in a multicenter study. The results of one older meta-analysis suggest that immunomodulatory treatment improves LV function in patients with symptoms longer than 6 months [96]. More recent meta-analysis of Lu et al. from 2014 evaluated the results of nine studies (covering a total of 342 patients treated with immunosuppression and 267 treated with conventional therapy) and showed that immunosuppressive therapy does not affect mortality or the need for heart transplantation, but favorable effect on improvement of LV systolic function was apparent. Conclusion of this study was that immunosuppressive therapy may be considered as an adjunct to conventional treatment, if this is not effective [97].

According to a majority of experts, but based on data from a single study, viral presence in the myocardium is associated with the absence of a positive response to immunosuppression (data from 17 patients, but only one was positive for PVB19) [69, 95]. Viral presence in the myocardium has not been determined in Myocarditis Treatment Trial (with neutral effect of immunosuppression) nor in the Polish study (with positive effect of immunosuppression on echocardiographic parameters), which makes the situation in this regard even more confusing [79, 98]. In the TIMIC study [77], worsening of echocardiographic parameters was observed in patients in the placebo group. Indeed, this is in contradiction with the results of other studies, including our own experience [22, 37, 79]. Because of this, CZECH-ICIT study was initiated with the ambition to bring more light to the uncertainties in the use of immunosuppressive therapy in myocarditis [99]. Recruitment of patients in the study is still in progress and the results are expected in coming years.

Treatment with intravenous immunoglobulins has a logical theoretical basis, which was confirmed in several small studies with quite favorable results [100, 101], but the largest multicenter trial by McNamara et al. showed no profit versus placebo [102]. Therefore, the administration of immunoglobulins is not currently considered as routinely indicated [2]. Similarly unclear is the position of immunoadsorption, where some studies have shown little effect on improvement of LV function, reduction of biomarkers levels, and retreat of inflammatory changes in the myocardium [5, 103, 104]. However, until these subtle data are confirmed by other studies, neither treatment could be recommended [2].

**Figure 4:**
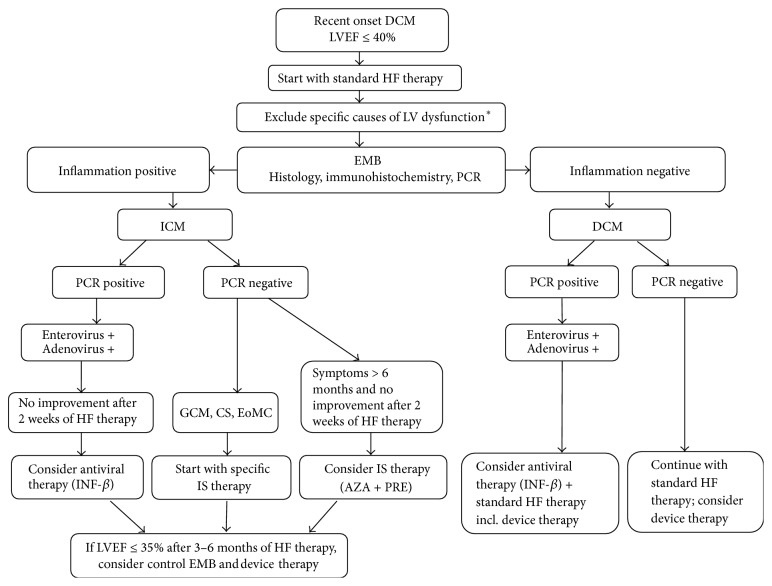
Diagnostic and therapeutic algorithm in suspected inflammatory cardiomyopathy. DCM: dilated cardiomyopathy, LVEF: left ventricle ejection fraction, LV: left ventricle, HF: heart failure, EMB: endomyocardial biopsy, ICM: inflammatory cardiomyopathy, PCR: polymerase chain reaction, GCM: giant cell myocarditis, CS: cardiac sarcoidosis, EoMC: eosinophilic myocarditis, INF-*β*: interferon-beta, and IS: immunosuppressive. ^*∗*^Specific causes of LV dysfunction: coronary artery disease, valvular disease, toxic causes (alcohol, drugs, and chemotherapy), tachycardia-induced cardiomyopathy, and endocrine disorders.

In the field of antiviral treatment, the published data are somewhat controversial as well. Administration of common antiviral drugs is possible, but there is no evidence about their actual effect. Theoretically, this treatment could be justified in the first phase of a disease associated with viral replication, but in clinical practice myocarditis is usually detected later in the second phase, when the administration is likely to have little benefit. It was proven that interferon-beta treatment removed enteroviruses and adenoviruses of the myocardium and in some studies was shown as beneficial [105]. In other more common types of viruses such treatment is unfortunately less efficient. That is probably the reason why the results of other studies with higher proportion of PVB19 were not so optimistic [106]. However, according to German authors, interferon-beta therapy may be at least in the case of enteroviruses associated with long-term prognostic benefit [29]. In PVB19 infection, telbivudine therapy is currently tested and we have to wait for the results. For some other rare agents, such* Borrelia burgdorferi*, antibiotic treatment is considered to be indicated, although the data from placebo-controlled studies are missing and also the results are not unequivocal [7, 8]. Algorithm with the proposal of therapeutic decisions based on knowledge of EMB result is shown in Figure 4.

## 5. Conclusion

The diagnosis of myocarditis and inflammatory cardiomyopathy remains highly complex and challenging despite the great expansion in diagnostic methods. Beside careful anamnestic data and physical examination, a comprehensive diagnostic approach using a range of noninvasive as well as invasive methods is required, together with highly sophisticated laboratory facilities. The most important noninvasive diagnostic method is cardiac magnetic resonance imaging, but endomyocardial biopsy still remains the gold standard. Standard therapy of inflammatory cardiomyopathy is based on the recommendations for the treatment of heart failure or arrhythmias; specific therapies may be indicated only with known results of EMB. Evidence for the therapeutic recommendation is not entirely convincing, and therefore individual assessment of each specific case and experience of the attending physician plays an important role in treatment decision. It is obvious that without carrying out large multicenter randomized prospective trial our therapeutic decisions will fall short of the requirements of evidence based medicine. A considerable effort is still ahead to reach comparable level of knowledge in the field of myocarditis and inflammatory cardiomyopathy to other areas of cardiology, where we have both clear and proven diagnostic criteria and also clear and robust data-based therapeutic recommendations.
